# Providing Effective Environmental Enrichment to Pigs: How Far Have We Come?

**DOI:** 10.3390/ani9050254

**Published:** 2019-05-21

**Authors:** Heleen van de Weerd, Sarah Ison

**Affiliations:** 1 Cerebrus Associates Ltd., The White House, 2 Meadrow, Godalming, Surrey GU7 3HN, UK; 2 World Animal Protection, 222 Grays Inn Road, 5th Floor, London WC1X 8HB, UK; sarahison@worldanimalprotection.org

**Keywords:** environmental enrichment, farming, pigs, sows, welfare, barriers to implementation, USA, China, EU

## Abstract

**Simple Summary:**

The welfare of farmed pigs can be improved by modifying their environment with bedding, substrates, or objects, so that they can perform more of their pig-specific behaviours. Scientific knowledge on effective enrichment for pigs is not necessarily reaching farms and this paper provides an overview of this issue in the three largest global pork producing regions. In the USA, enrichment has not yet appeared on farms, except when required by higher welfare farm schemes. China hardly has any animal welfare legislation and food safety concerns restrict the use of enrichment on farms. Providing pig enrichment is required by law in EU Member States. In practice, enrichment is not always present, or is unsuitable or inadequate. Other risks to animal welfare include inadequate presentation, location, quantity and size, and maintenance of enrichment. Improvements can be made by applying principles from other fields of behavioural science; welfare knowledge transfer and training to farms; highlighting the economic benefits of effective enrichment; increasing pressure from the financial sector; using novel drivers of change, such as public benchmarking. The poor implementation of scientific knowledge on farms suggests that the industry has not fully embraced the benefits of effective enrichment.

**Abstract:**

Science has defined the characteristics of effective environmental enrichment for pigs. We provide an overview of progress towards the provision of pig enrichment in the three largest global pork producing regions. In the USA, enrichment has not yet featured on the policy agenda, nor appeared on farms, except when required by certain farm assurance schemes. China has very limited legal animal welfare provisions and public awareness of animal welfare is very low. Food safety concerns severely restrict the use of substrates (as enrichment) on farms. Providing enrichment to pigs is a legal requirement in the EU. In practice, enrichment is not present, or simple (point-source) objects are provided which have no enduring value. Other common issues are the provision of non-effective or hazardous objects, inadequate presentation, location, quantity and size or inadequate maintenance of enrichment. Improvements can be made by applying principles from the field of experimental analysis of behaviour to evaluate the effectiveness of enrichment; providing welfare knowledge transfer, including training and advisory services; highlighting the economic benefits of effective enrichment and focusing on return on investment; increasing pressure from the financial sector; using novel drivers of change, such as public business benchmarking. The poor implementation of scientific knowledge on farms suggests that the pig industry has not fully embraced the benefits of effective enrichment and is still a long way off achieving an enriched pig population.

## 1. Introduction

The scientific investigation of environmental enrichment originated in the 1960s [1], with a gradual shift in focus from animals in the laboratory, to the zoo, to the farm and to companion animals, as well as a shift in focus on increasing naturalistic behaviour and reducing the incidence of behavioural problems to enhancing welfare.

The term environmental enrichment has been used widely, and the term is often used for anything that gets added to a captive environment. However, from a scientific point of view, it should only be applied to situations where environmental modifications have enhanced the performance of strongly motivated species-specific behaviours or have led to the expression of a more complex behavioural repertoire [2].

Foundational research on environmental enrichment for farmed pigs has focused on the features of housing systems that meet the key behavioural needs of pigs [3]. More recently, applied welfare science has highlighted the main characteristics of effective enrichment. At the same time, awareness of farm animal welfare issues amongst stakeholders such as consumers and food animal producers has increased [4,5,6].

Furthermore, public pressure for reforms in farming systems also increased because of the risks associated with animal production and international animal trade [7]. Some of these risks have been brought to public attention through high profile animal-health related crises such as BSE (Bovine spongiform encephalopathy), foot-and-mouth disease and African Swine Fever, sometimes coupled to food safety scandals (e.g., clenbuterol fed to pigs in China [8]).

Mellor and Webster [9] describe the process by which societies adapt to increasing knowledge about animal welfare issues as a journey. Countries, regions, businesses and individuals travelling on this journey reach the destination of improved animal welfare at different rates and in different ways. The drivers on this ‘journey’ will vary in different global regions, but a broad categorisation is possible [10,11]:

- Regulation;
- Concern about animal welfare from consumer/citizens, actioned via non-governmental organisations (NGOs);
- Welfare standards and private animal welfare initiatives (assurance schemes);
- Agri-sector business innovation and business engagement, for example via Corporate Social Responsibility.

Scientific knowledge of animal behaviour and welfare has underpinned progress in each of these categories of driver [12]. While these drivers have been defined for animal welfare in general, they will equally apply to pig welfare.

This paper provides an overview of the progress towards the provision of enrichment that satisfies pig-specific needs (‘effective enrichment’) in the main global pig-producing regions. This information will illustrate the apparent mismatch between the scientific understanding of what constitutes good and effective enrichment for pigs and the practices on pig farms around the world. Some of the main barriers to providing effective enrichment and possible ways to overcome these will be highlighted and discussed.

## 2. Science of Pig Enrichment

The main aim of environmental enrichment has been defined as to improve the biological functioning of captive animals [2]. It is therefore important to have detailed knowledge of the particular species concerned when designing and implementing enrichment strategies [13]. Domesticated pigs still express very similar behaviour to their wild ancestors and this has implications for pigs in our farming systems and the behaviours we try to satisfy with the provision of enrichment [14].

The first main goal of enrichment for pigs is to enhance barren living conditions associated with intensive production systems and to provide a suitable outlet for performance of their species-typical behaviour, thus enhancing their welfare. The second goal is to use enrichment as a tool to manage undesirable and damaging behaviours, such as tail biting, to prevent escalation of this behaviour. These two goals are linked as the risk of pig manipulation behaviour is reduced when pigs have a suitable outlet for their species-specific behaviour [15].

In the case of tail biting, enrichment is an important ‘emergency’ tool, especially when early signs of tail biting are observed (e.g., tail posture [16]). In such situations, additional enrichment (e.g., salt and mineral lick stones, substrates such as Lucerne/alfalfa) can be provided to prevent escalation of the undesirable behaviour, and it is important to apply enrichment that is not used on a daily basis. When novel enrichment is provided, it may reinvigorate the response to and engagement with the enrichment, while animals may have habituated to enrichment that is continually present in the pen [17]. It should be noted that tail biting is a multi-factorial issue [18,19], with many factors contributing to the risks. Enrichment is not the only tool for managing tail biting, and success of the intervention depends on the underlying causes of the specific outbreak.

The prevention of tail biting in growing meat pigs has been the focus of much research on enrichment. These animals are most at risk of this behaviour [1,20]. However, this leaves several knowledge gaps with regards to effective enrichment for other types of pigs. While there is a large body of literature on pre-partum nest building in sows and the use of nesting materials [21], there is much less literature on enrichment for both sows and piglets during the lactation phase. The review by Vanheukelom et al. [22] found beneficial effects on the welfare of both piglets and sows, by providing opportunities to engage in explorative behaviour, nest-building and social interactions and improving maternal responses. These positive effects can also extend into the growing phase [23]. 

Research on effective enrichment for gestating sows and especially gestating sows in group housing is even scarcer. It is important that these animals are studied separately as their motivation to interact with enrichment is expected to be different from that of growing pigs due to their nutritional status combined with their strong social dominance relationships [24]. Indeed, Stewart et al. [25] found that sham chewing (a stereotypic behaviour that is related to their restricted diets) of pregnant sows in dynamic groups was not reduced by the provision of straw racks, although there was a reduction in pen-directed exploratory behaviour. Aggressive activity may occur at locations of highly valued resources, such as enrichment [26]. Limited access to valued enrichment can result in greater aggression towards subordinate sows and higher stress levels in those animals [27]. Other studies also found increased aggression [25], although, in some studies, aggression remained unchanged after the provision of enrichment [28].

Enrichment for breeding boars has hardly received any attention from the scientific community [1]. These animals are mostly housed individually with limited social contact, and are also (outside Europe), kept in sow stalls with very limited movement and occupation opportunities. The behaviour and housing requirements of mature boars are poorly understood [29] and these animals also require some form of enrichment or a floor covered with a substrate bedding. 

### 2.1. Characteristics of Effective Enrichment

Research has produced a wealth of knowledge on what constitutes effective environmental enrichment [30,31,32] with associated information on how enrichment can be best applied in practice [18,33]. Functional and effective enrichment needs to meet a series of specific characteristics (Table 1) that will enable pigs to show certain behaviours while interacting with the enrichment. This is linked to the method of provision.

Most types of particulate substrates incorporate the characteristics as listed in Table 1 and are therefore regarded as effective enrichment [35] when provided as bedding (covering the floor area)—and are well maintained—or provided in smaller quantities in racks or dispensers [36]. 

If bedding cannot be provided (for example due to incompatibility with slatted floor systems or unsuitable geographical climate conditions), producers often resort to providing objects, such as simple chains, plastic pipes or commercially available pig ‘toys’. These types of point-source objects (objects limited in size, and restricted to a single location in a pen [1]) may be quick and easy to implement, but their effectiveness is often limited. They may, for example, not prevent tail and ear biting [15,37].

While many studies have evaluated the effect of individual point-source objects, findings are not always consistent or as predicted, due to the range of possible combinations of variables being tested (e.g., method of presentation, location in pen, quantity of material in relation to group size, age and type of pig, etc.) and the confounding of several of these characteristics [17,32].

The main issue with many point-source objects is that the behaviour seen as a result of the interaction is often short-lived and, for pigs, it is mainly intrinsically reinforced (such as exploration). In the absence of a relationship between behaviour and an external consequence such as food, interaction with enrichment is intrinsically motivated [17], so a pig will lose interest following exploration of an object when it has lost its novelty [30,38] and habituation occurs. Conversely, effective enrichment has longer-lasting effects as it provides extrinsic reinforcement. For example, the performance of (intrinsically motivated) exploratory behaviour directed at an enrichment object would be extrinsically reinforced if the behaviour results in finding food items or substrates that can be ingested.

As long as the enrichment continues to provide meaningful (extrinsic) reinforcement, it will retain enrichment value on subsequent exposures [17], for example after an animal has had a period of rest. Rotating enrichment objects, altering the appearance or properties of the items, and increasing the difference between the item and the rest of the animals’ environment, as well as providing ingestible rewards on a variable schedule of reinforcement will help to maintain a level of response and slows down the rate of habituation (see [17] for an in depth discussion of intrinsic and extrinsic exploration in the context of enrichment; see also [39]). 

Knowledge of the motivation for the performance of a behaviour is very important for predicting the effectiveness of the enrichment. Selecting effective enrichment does not only rely on the characteristics (Table 1) and issues outlined above. There are further aspects to consider for an enrichment programme to be successful. Van de Weerd and Day [1] proposed a framework (building on [2]) that includes health, practical and economic aspects for the evaluation of the success of enrichment. The four criteria are:

1. Enrichment should increase species-specific behaviour;
2. Enrichment should maintain or improve levels of health;
3. Enrichment should improve the economics of the production system;
4. Enrichment should be practical to employ.

Networks led by farmers and the industry can generate practical and effective solutions to animal welfare problems, as they will know the problems to solve and they will know how to design a solution that is meaningful for the specific farming community [40]. However, innovations may be driven by improvements in productivity or profitability, rather than by animal welfare driven and enrichment solutions may not always fulfil all the criteria for effectiveness and can be expensive. Creative producers can make their own enrichment objects by using materials that are easily available on farms (e.g., feed sacks) and by following the principles as outlined in this paper (see also more practical guidance in [34]). 

## 3. Pig Enrichment in Global Practice

Global pork production has increased 4-fold over the last 50 years and is expected to continue growing during the next three decades due to population growth (to 8.6 billion in 2050) and an increased per capita consumption [41]. 

The top pig-producing regions globally are China (47% of total world production), Western Europe (20%) and the USA (10%) (FAO data from 2016, published in [42]). These three regions represent different stages on the ‘animal welfare journey’ (see introduction [9]) and, therefore, on their path to enriched pigs. The next section reviews the status of enrichment provision for pigs in these global pork producing regions and describes which of the drivers towards higher welfare play a role locally.

### 3.1. USA

The USA’s 10% share of global pork production [42] accounted for 6% of US agricultural sales (in 2012 (USDA Census Highlights, Hog and Pig farming (accessed on 26 March 2019)). The USA is also the second largest exporter of pork products (FAOSTAT, 2016, Crops and livestock products (accessed on 26 March 2019)). The consumption of pig meat in the USA was decreasing for a short period (2009–2012) but has increased again in recent years and is projected to remain stable with a slight increase in years to come (OECD Meat Consumption data (accessed on 26 March 2019)). These projections may change depending on the development and adoption of alternative proteins (plant-based protein, edible insects, clean or cultured meat) in US diets [41,43]. 

There is no federal legislation to protect the welfare of farm animals during rearing [44]. Regulations under the Animal Health Protection Act give the Department of Agriculture general authority to broadly protect farm animal health, but not specifically welfare. There is, however, some federal legislation to protect farm animals during transport and slaughter (although poultry are excluded). This includes the 28-h rule for transport to provide rest, feed and water every 28 h, but there is no mechanism for monitoring, meaning enforcement is problematic. The Humane Slaughter Act 1958 dictates that farm animals are handled and slaughtered in a humane way (also excludes poultry) and the Federal Meat Inspection Act provides inspection of handling and slaughter. 

At the state level, only 16 of 50 states include livestock in anti-cruelty legislation [45,46]. Thirty states have additional humane slaughter legislation [44]. Considering all animal protection legislation, the top three states are California, Oregon and Massachusetts, with Mississippi, North and South Dakota at the bottom [47]. Through ballot initiatives, 12 states have existing or planned legal limitations on the use of close confinement systems (sow gestation crates, veal crates, and/or battery cages for laying hens), including 10 states limiting the use of gestation crates [45,46]. Additionally, two states (California and Massachusetts) limit the sale of products from close confinement systems. California started this trend in 2010 (Proposition 2, prohibiting the sale of caged eggs), recently strengthened with Proposition 12 (limiting confinement for veal calves, pigs and egg laying poultry). 

Pigs are mainly raised on intensive farms. The majority of these are large scale, with two-thirds of pigs housed in farms of over 5000 and 90% on farms with over 2000 pigs (Census of Agriculture United States, 2012 (accessed on 26 March 2019)). Intensive systems typically include total confinement (enclosed, mechanically ventilated buildings) with fully slatted floors and no substrate/bedding or enrichment.

Pig enrichment has not yet featured on the political agenda, nor on the agenda of civil societies. The use of enrichment on farms is sporadic and mainly appears on ‘niche farms’ and/or those operating under certain third-party audited farm assurance schemes (Animal Welfare Approved; Global Animal Partnership 5-step program (Step 2 and above); American Humane Certified; Certified Humane (all accessed on 26 March 2019)).

‘Niche’ production for alternative marketing avenues is gaining interest from the US consumer and producer alike [48,49]. These production systems are typically characterised by outdoor and/or bedded systems, heritage breeds, no sub-therapeutic antibiotics or growth promotors, vegetarian feed and a small- to mid-size family farm production setting. They provide a higher standard of welfare for the pigs, a quality product for the ethical consumer with a market premium (often covered by third-party certification schemes) and personal satisfaction for the farmer [48,49].

As part of the ‘We Care’ initiative, the National Pork Board’s voluntary industry-led scheme, Pork Quality Assurance (PQA) Plus (Pork Quality Assurance (PQA) Plus (accessed on 26 March 2019)), aims to promote food safety, public health, a safe working environment and animal well-being. The scheme has two components, individual certification following an education program and site status achieved after farm site assessment by trained assessors. However, the well-being sections of the individual and site assessments exclude the provision of enrichment [50,51].

Interest in the provision of environmental enrichment for pigs in conventional confinement systems currently focuses on its utility to improve production performance. This includes minimising unwanted inter-pig aggression, especially at the point of regrouping, damaging behaviour such as tail, ear and flank biting and stereotypic behaviour [26,52,53]. This interest is in its infancy, meaning peer-reviewed research papers are scarce. However, the National Pork Board’s 2019 call for research proposals includes “Review the benefits of enrichment and their impact on preventing aggressive and damaging behaviors in various housing environments and on overall production.” In addition, the USDA has environmental enrichment for gestating sows, farrowing sows and piglets and weaner, grower and finisher pigs built into the current work plan [Jeremy Marchant-Forde, Personal communication, 2019]. Iowa State University has a project underway to create and test (in a research and commercial setting) a novel pig dietary enrichment to improve survivability during the weaning transition [Anna Johnson, Personal communication, 2019].

Non-governmental organisations (NGOs) actively campaign on behalf of their supporters to improve animal welfare. These organisations can influence public opinion via activities such as public awareness-raising campaigns and targeted direct action, but also dialogue with industries and regulatory bodies, all with varying levels of impact [54,55]. Combining legislative work with undercover investigations, litigation and corporate engagement, NGOs have played a role in transitioning the pork industry away from gestation crates over the last decade [55]. A significant aspect to this success in the US has been through corporate engagement, raising the gestation crate issue with major pork buyers, including restaurants, grocery stores, fast-food chains, and others in the retail sector [55]. This has now extended to other pig welfare issues including the provision of enrichment (see for an example [56], although reporting shows that only small numbers of pigs receive enrichment and that the provided objects—balls, chains and toys—do not meet all the characteristics of effective enrichment as per Section 2.1). The future outlook is focused on providing pigs with effective enrichment and to better understand the challenges producers face in an uncertain market in order to invest in improved housing conditions for pigs.

A lack of federal legislation protecting farm animal welfare during rearing, as well as few and inconsistent provisions of state legislation, are barriers to improving pig welfare [44]. In addition, concerns for biosecurity and manure management in relation to the use of environmental enrichment [26], as well as a lack of recognition of the need for enrichment by the industry, is likely to further hamper progress. Due to the nature of conventional systems in the US, it is unlikely that enrichment will fulfil the characteristics of effective enrichment without significant infrastructure changes. Consumer-driven improvement in food company animal welfare policy, implementation and monitoring may be the most promising route to achieving effective enrichment in the USA. However, information on public attitudes towards farm animal welfare and perceptions of current and proposed future animal production systems is needed to ensure future systems reflect the public’s ethical values about the treatment of animals [57].

### 3.2. China

China accounts for almost half of the world’s pork production and pork was, and still is, the predominant staple meat for Chinese consumers, with around 700 million pigs slaughtered annually—one for every two Chinese people [58]. In addition to being almost the biggest pig producer, China is also the biggest importer of pig meat and shows the biggest increases of imports in the last 10 years [42].

Rural families traditionally raised pigs as part of small-scale farming operations, and these are now increasingly being replaced by larger-scale farms that use grain-based feeds [59]. As a consequence of active government policy (since 2007), the average pork enterprise size has increased towards industrialized production, with large farms taking advantage of scale economies in accessing better genetics, addressing quality issues and mechanizing to replace labour.

There are societal and cultural awareness barriers to improving animal welfare, and a large proportion of Chinese consumers view animal welfare (e.g., improve the rearing conditions) as part of the food safety of animal-derived products [60]. However, there are signs that society is increasingly aware of the concept of animal welfare and of the need to establish laws to improve animal welfare *[ibid.]* and, more specifically, of the need to improve welfare during transport and slaughter [61]. Animal welfare awareness is higher and more developed in globally-oriented cities such as Beijing and Shanghai with the highest income, education, and greatest exposure to modern lifestyles and information [62].

With regards to animal welfare legislation, the Chinese government has enacted some laws regarding laboratory animals, zoo management and practising veterinarians and these provide some level of general protection to animals. In addition, there is some legislation relevant to farm animals, specifically regarding transport and slaughter. However this is mainly based on food safety concerns that touch on some animal protection [63].

The Chinese Veterinary Medical Association (Chinese Veterinary Medical Association (CVMA, accessed on 4 March 2019), supervised by the ministry of Agriculture, has a branch that deals with animal welfare (Animal Health and Welfare Association). The CVMA is developing non-binding guidelines on the welfare of animals (including farmed). Despite minimal legislation on animal welfare, the Chinese Government approves activities on animal welfare. A good example is the International Cooperation Committee on Animal Welfare (International Cooperation Committee on Animal Welfare (ICCAW), accessed on 4 March 2019), a non-profit organization approved by the Ministry of Agriculture and Rural Affairs that facilitates communication within the China livestock industry and international welfare organisations such as World Animal Protection and Compassion in World Farming. 

The ICCAW (together with stakeholders) has published basic welfare standards for the main farmed species: pigs, chicken (laying hens, broilers), beef cattle and sheep [64]. The standards include guidance on enrichment (standard 5.7) referring to adequate manipulable materials (in line with scientific knowledge) to prevent abnormal, and allow, natural behaviour. These standards provide the first set of (voluntary) guidelines for pig welfare in China.

Independent of these activities, progressive businesses develop their own welfare programs. The first animal welfare issues to address are reducing the time pregnant sows spend in sow stalls and abolishing these altogether and move to group housing as well as to provide enrichment to all pigs. Collaborative projects have developed at an academic level [65] and on a more practical on-farm level [66]. 

Despite the existence of the ICCAW pig guidelines that mention enrichment, there are limitations as to what can be achieved on farms. Food safety is currently one of the main concerns of the Chinese pork consumer due to a string of food poisoning incidents in recent decades (see [67] for overview). This has had repercussions as consumers seem to prefer pork from industrialised farms as they associate that with constant quality and high food safety levels of the final product *[ibid.]*. 

This focus on food safety poses a main constraint on what materials farms are willing to use, as it severely restricts the use of substrates as enrichment materials, unless the farms know that these materials originate from safe sources. The consequence of this limitation is that when enrichment is provided, it often consists of a simple point-source object that does not necessarily meet all the requirements of effective enrichment (see Section 2.1).

Progress on farms can also be hampered due to a low awareness of animal welfare (both as a concept and on how this works in practice), and staff often have low technical skills, coupled with a high staff turnover. There is no effective management and infrastructure to collect information of on-farm practises at the government level [68], which will make encouraging, implementing and enforcing higher welfare practises very difficult. Another challenge for companies is that the marketing of products that are produced under higher welfare conditions is difficult with low consumer awareness of animal welfare. However, some consumers believe that when animals are treated well, their products taste better than regular products and this may provide marketing opportunities.

### 3.3. European Union

The EU is the second largest meat producing region worldwide [42]. Within the EU, pig meat represents 9% of agricultural output. While pig meat production has remained stable over the years, the total number of pigs has decreased since 2004. This reflects efficiency gains in pig farming (larger pig farms with more sows) despite stronger regulatory constraints (e.g., on welfare, such as group housing for sows, leading to increasing costs per sow) [69].

Compared to the USA and China, the European consumer appears more aware of farm animal welfare issues and also expresses clear views on this. In the most recent Eurobarometer survey [6], the majority of EU citizens interviewed (82%) believed that the welfare of farmed animals should be better protected than it is now, and 64% agreed with the statement that they would like to have more information about the conditions under which farmed animals are treated in their respective countries.

Legislation is the most common type of policy instrument used by the EU to achieve minimum welfare standards for farm animals. The aim of the policy is to spare animals all unnecessary suffering in three main phases of their lives: farming, transport and slaughter and to prohibit some of the most inhumane aspects of intensive, industrial livestock production [70]. The EU’s approach to animal welfare legislation reflects changes in scientific understanding, changes in animal management practices, socio-economic studies and increasing consumer concern for animals (often for ethical reasons) and public expectations [71] and this has led to legislated animal welfare standards that are the most stringent in the world. 

Pigs are protected under a specific directive (directives are a policy instrument in which the results to be achieved are binding, but each Member State’s national authorities can choose the form and methods to get these results) that requires the provision of enrichment to all pigs. The relevant sections of the Pig Directive (2008/120/EC) that refer to enrichment are shown in Table 2. 

Legislation is not the only pathway to higher welfare. Private or voluntary animal welfare initiatives such as (welfare) farm assurance schemes (Notable examples are Soil Association organics and RSPCA Assured in the UK, Beter Leven in The Netherlands, Neuland in Germany, KRAV organics in Sweden and Label Rouge in France) promote good husbandry practice on farms and drive production standards towards higher welfare [73]. These schemes also increase compliance with animal welfare legislation as they are based on legal standards, coupled with regular audits [74,75]. Animal welfare assurance schemes (and associated food labelling) allow consumers to avoid the need to reflect on their food choice and the animals from which such products were derived, by delegating responsibility for issues such as animal welfare onto other actors, such as the state, supermarkets or brands [76]. Although in actual fact, consumers are often ignorant about the animal welfare standards within a particular scheme [77].

The European legal landscape, combined with animal welfare assurance schemes and individual business initiatives, provides a wealth of practical guidance for higher welfare in intensive pig production. There has been much recent activity to better implement the Pig Directive 2008/120/EC, with a focus on moving away from tail docking [78,79]. These studies show that, in countries that have banned tail docking (Finland, Sweden [80]), producers mostly provide straw and about 50% of the growing pigs in the UK are kept on straw [81]. Additionally, producers from several other countries are finding solutions to rear pigs with tails that involves providing effective enrichment. However, to be successful, this also requires a substantial change of management, with careful animal care, lower stocking densities, improved air and water quality, and the provision of a stress-free environment, which most farmers with existing systems find very difficult to achieve [78]. Furthermore, as outlined in Section 2, enrichment should not only be provided in systems that rear pigs with intact tails. 

So, despite some success stories, the reality is much bleaker. Producers do not always provide enrichment and, if they do, it is mainly provided to intensively farmed weaned and growing/fattening pigs and mostly achieved with the provision of ‘toys’ (point-source enrichment-objects). The lack of providing material for manipulation to pigs constitutes a regular non-compliance with the Pig Directive [78]. These infringements have been highlighted following several inspection visits by the Food and Veterinary Office (now called Health, Food Audits and Analysis department of DG For Health and Food Safety) in several Member States (e.g., 82 FVO mission reports on animal welfare in the period 2005–2010 [82,83]. More recently, an EU Health and Food audit in Italy highlighted a lack of guidance on enrichment provision (against a background of managing risks to tail biting) [84]. The EU highlighted its intention on improving enforcement of legal requirements in its Strategy for the Protection and Welfare of Animals 2012–2015 [85]. 

Animal welfare NGOs have also focused on this issue, for example by publishing a compilation of photos taken from publications and websites from the EU pig industry [86], showing the absence of enrichment. While this is neither a very structured nor scientific approach, it does highlight how enrichment is regularly lacking as it should be visible in every pig pen, in the same way as feeders and drinkers are. When enrichment is provided, it is often an indestructible simple object which has no enduring value to the pig. 

### 3.4. Common Deviations on EU Farms

#### 3.4.1. Non-Effective or Hazardous Objects

Non-effective objects are very often provided. These objects do not meet the needs of pigs (as defined above) and can pose a hazard as they are unsafe (e.g., ingestible metal parts in car tyres [35]). Many studies have confirmed that objects such as chains, plastic pipes and balls or car tyres should not be recommended for long-term use, as they are not effective enrichment (e.g., [87,88]), do not meet the criteria of effectiveness and can quickly lose their novelty factor [1]. Composite objects are also often provided, e.g., by adding balls, pipes or pieces of hard wood to a metal chain. However, pig experts agree that trying to improve a metal chain in this way, only marginally improves welfare, remaining well below what they consider acceptable enrichment [81]. 

There is also concern that the manipulation of inadequate materials causes frustration when pigs try to interact with them (e.g., trying to bite in a ball or wooden log that is too wide for their mouths) [Van de Weerd, Pers. obs.]. Despite this, the use of such objects is still fairly widespread on farms, e.g., small balls on chains are prevalent in the Netherlands and Germany, while barren chains (without added destructible objects) appear to be prevalent in France and Belgium [81], and chains and plastic objects on farms in the UK [82]. Furthermore, the provision of materials such as solid wooden blocks, which are widely provided and perceived to be accepted as sufficient to comply with the EU Directive, can result in high levels of tail biting, especially if tail docking is not also used [89].

There are signs of progress, for example the debate in the Netherlands has moved on from being focused on implementing enrichment per se to the type of enrichment provided, with the government bodies recently announcing that they will enforce measures against inadequate enrichment such as a ball on a chain [90]. 

#### 3.4.2. Inadequate Presentation

Other common mistakes are that (non-effective) objects are presented in the wrong way, such as loose on the floor (e.g., plastic canisters or balls) or hanging too high for pigs to reach (pigs are not able to lift their heads very high due to the anatomy of their neck). Although species-typical pig exploration is mainly directed at the ground level, suspending and attaching (with objects at eye or floor level) are favoured enrichment characteristics, because attachment prevents objects lodging in corners, becoming stuck behind feeders or getting pushed into neighbouring pens, out of reach [30,32,91].

#### 3.4.3. Inadequate Location

Point-source objects as well as substrate racks or dispensers may be presented in the wrong location, with the only consideration being ease of maintenance (e.g., refill) for animal care takers. Objects suspended over the areas where most pigs sleep will lead to disruption of sleep patterns [1]. Enrichment should therefore be provided close to (but not in) the areas that are used for eating, drinking and elimination. This means that pigs who are active (move, eat, drink or manipulate enrichment) do not disturb sleeping pigs and enrichment does not get soiled with manure.

The location of substrate racks for group-housed (pregnant) sows needs careful consideration, especially in dynamic groups, as there will be high levels of activity near the racks, especially immediately post-feeding. Enrichment objects can be used to decrease activity around an electronic feeding station that normally attracts queuing sows, by leading animals away from the waiting and feeding station areas. Additionally, enrichment provided in a corner where only a small number of pigs can gain access can cause problems such as competition, aggression and displacements (see next section).

#### 3.4.4. Inadequate Quantity/Size

If point-source enrichment is too small or of limited quantity, this will restrict availability [92], especially when grouped pigs synchronise their interactions with enrichment [36,93]. Limited accessibility to enrichment materials or objects may lead to social competition, aggression or restlessness and the redirection of exploration behaviour to pen structures or pen mates [93]. Distributing enrichments throughout a pen will help to reduce any negative effects of social status in sows [27] and reduce displacements [94]. Increasing object size will allow more pigs access simultaneously, but ease of access is also related to group size. 

The size of enrichment objects should also be adjusted to the age and size of the pigs that they are presented to. If pigs are not able to adequately bite enrichment (grab between their jaws), they will be less able to chew, manipulate and possibly ingest, making the object less effective (see Table 1). This could explain why wooden logs with a diameter of 10 cm appeared less suitable as enrichment for weaner pigs (around 6.5 kg) compared with chains [95], and in comparison with wooden logs with a diameter of 10 cm provided to grower pigs of around 20 kg (and upwards) [23]. However, the method of attachment (in a wall-mounted bracket, or on loose chains), also differed between these two studies.

Systematic research into the number and placements of point-source objects in relation to group size would be valuable to elucidate these factors [1].

#### 3.4.5. Inadequate Maintenance

Point-source objects are often put in a pen before a new batch of pigs arrive, but are subsequently not maintained, cleaned or renewed (if destroyed). Substrate bedding needs to be topped up daily to stay fresh and clean. It remains unclear whether pigs are deterred by soiled enrichment objects, an issue first raised by Blackshaw et al. [91]. Recent studies confirm lower interaction with logs presented on the floor, versus a hanging log, possibly caused by soiling with faeces [87]. However, the study on this issue by [96] found that cleaning (plastic and rubber) objects did not affect the pigs’ interaction with them.

There are many aspects to consider with regards to optimal renewal or replacement rates, as long as the enrichment continues to provide meaningful reinforcement, it will retain enrichment value [17]. The risk of an object not retaining its enrichment value is illustrated by the pigs in a study by [87] who lost interest in non-effective enrichment objects (poplar wooden logs presented on chains or on the floor) quickly over a period of 6 weeks, whereas levels of tail and ear biting stayed at similar levels, thus increasing the risk of a tail-biting outbreak at some point.

Pigs can retain a memory of objects and therefore it is recommended to have a continuous rotation of point-source objects between pens, whereby the object exposure is limited to not more than two days and the same object is not introduced again until five days have passed, as this may help to preserve the exploratory value (novelty) of the objects rotated [97].

## 4. Main Barriers (Globally) and How to Overcome These 

The pace and uptake of change in farm animal welfare is slow despite the demonstrable benefits of changes to the animals concerned. This suggests significant and persistent barriers to the uptake of practically applicable knowledge [98]. The overview of the situation in the main global pig-producing regions shows that this is clearly the case for enrichment for pigs. This barrier and other potential barriers, as well as possible ways to overcome these, will be discussed in this section.

### 4.1. Knowledge Transfer and Training

Despite the existing body of applied animal welfare knowledge on pig enrichment, there is still a need for more fundamental knowledge by applying theoretical frameworks. Tarou and Bashaw [17] describe several well-studied principles from the field of experimental analysis of behaviour and learning theory [99] as applied to enrichment for laboratory and zoo animals. Their approach is very useful for evaluating the short- and long-term effectiveness of enrichment and testing their predictions remains crucial for understanding and increasing the efficacy of enrichment programs for all captive animals, including farm animals.

There is an expectation that those who work with farm animals should know about animal welfare and animal behaviour issues, but there may still be a big gap in technical knowledge of farm staff, on pig-specific behaviours and on how motivated behaviour can channel into adverse behaviour, especially in restricted barren environments (e.g., the need to explore is channelled into tail biting [14]). Ultimately, influencing the behavioural attitude of farmers and animal caretakers can have a great effect on an animal’s well-being and attitudes can change with new experiences or information about the animals [100]. 

Opportunities for welfare knowledge transfer include training and the use of advisory and extension services. These services give farmers and other food-chain actors access to information and knowledge, and, in return, inform research communities of the circumstances of its application in farm practice [98]. This ensures feedback between knowledge generation and its implementation. Furthermore, effective information delivery is dependent on understanding the motivations of animal care-takers and farmers as this is a pre-requisite for their potential receptivity of advice [101].

A fundamental requirement for the success of knowledge transfer will depend on the availability of good resources on enrichment [18,33] and training facilities [102,103] and suitably skilled people who can assist with implementing this knowledge in practice and present information accurately and without bias. Successful approaches to delivering animal welfare messages may have to utilise less unidirectional strategies, and more collaborative and participatory approaches. Such a method is successfully used in China by the NGO World Animal Protection [66]. Participatory approaches are effective in identifying and recognising common problems and creating common strategies for addressing them. In doing so, they allow farmers to retain ownership and control over possible solutions and methods to achieve them [98].

### 4.2. Economics (Return on Investment)

Applied animal welfare science does not have a great track record in linking animal welfare outcomes to economic benefits and the focus of enrichment programmes has often been on costs (see, e.g., the costs of providing wood enrichment in [104]). However, there has to be an obvious return on investment for producers to adopt effective enrichment strategies, otherwise it poses serious barriers for implementation [1] and hampers the ability or will of the pig industry to develop innovative enrichment.

When farms implement enrichment, their biggest investment is often not so much the cost of the materials, but investment in staff time to manage extra work (e.g., installing and maintaining enrichment, monitoring animals [89] and investment in staff (animal welfare) knowledge and technical skills. These issues are closely related to suggested barriers to abandon tail docking [78]. The focus for progress has to be on the returns that high standards of animal care can yield. The costs and associated economic benefits of providing enrichment and reduce damaging behaviours such as ear and tail biting have so far been mostly studied.

The costs of tail biting have been assessed and range from an extra net cost of €18.96 for a victim of tail biting, impacting on the net margin of production (modelled data) [89], and up to 43% less profit per pig, due to carcass condemnation and trimming losses (slaughter house study) [105]. Furthermore, daily weight gain in tail-bitten growing pigs can be reduced by 1 to 3% [106].

Disease is a concomitant threat to animal welfare and business sustainability, but few studies have focused on the relationship between enriched environments and health issues or disease susceptibility. Some research shows the potential of studying this relationship. For example, straw bedding reduced the incidence of gastric lesions in growing pigs, reflecting either lower levels of stress as compared to barren housing or a positive effect of stomach content firmness [107]. Enriched housed pigs showed less stress-related behaviour and had reduced disease susceptibility to co-infection of PRRSV (Porcine Reproductive and Respiratory Virus) and *A*. *pleuropneumoniae* (associated with fewer lesions in the lungs, and a lower total pathologic tissue damage score) [108].

For group-housed pregnant sows there is even less scientific information on the relationship between the facilities in a group-housing system and infectious disease, although the fact that the lying and defecation areas are separated improves hygiene and decreases the intensity of oral contact with faeces [109].

There are also possible gains in terms of performance that need to be quantified, e.g., crate-born piglets raised with enrichment from birth can have improved growth and a modulated immune response compared to piglets from barren crates [110]. Future efforts should focus on quantifying the benefits of enrichment in relation to the improved performance of growing pigs [32], improved performance of sows (e.g., lower lameness levels, lower stress levels and stereotypies, improved pregnancies, ease of farrowing, shorter partus, increased maternal care), possibly leading to more pigs/sow/year; and possible improved piglet survival (e.g., coping with and adapting to weaning, reducing the growth check after weaning, e.g., [111]).

There is a clear role for applied ethologists together with agriculture economists to describe these economic benefits of higher welfare strategies (or adversely, the costs of low welfare). Mentioning economics raises the issue of who should pay for higher welfare production with the finger often pointing at consumers who should be willing to pay the increased costs [89] via a price premium for higher welfare products (see, e.g., ‘Heart Pig’ higher animal welfare brand (Denmark), with sows mainly in loose housing, growing pigs with 10% more space and not tail docked and with continuous access to straw in racks. The production costs are 7.9% higher (e.g., covering increased costs for more, straw, mortality, labour), rising from €1.41/kg up to €1.52/kg slaughter weight. These higher costs are covered by a price premium for the meat, approximately €0.17 per kilo [112]. However, we may have to think broader and re-invent the business model for higher welfare farming and re-think how welfare is valued in the supply chain, for example by applying similar methods as those applied to environmental monitoring services [113]. The participative process presented by these authors combines open innovation in idea generation, evaluation and the development of ideas and identification of a new core business model. Such a model should build on adequate compensation and incentives for producers that collect and contribute valuable animal welfare data.

### 4.3. Novel Drivers of Change

The commitments of food companies can be a major driving force to influence the welfare of animals, especially if the commitments expressed are translated into actual behaviour [11]. There is increasing pressure on food companies to include animal welfare as an area of focus within their business objectives, especially those that have animal products in their supply chain (e.g., food retailers, processors and food service businesses). 

Corporate Social Responsibility (CSR) is the principle instrument to communicate a company’s ethical and social commitments [114] and CSR statements are also an important component of branding, assuring consumers that due diligence requirements are being met and helping to retain customer fidelity [98]. Many companies have incorporated animal welfare statements within their social responsibility targets [11,115]. Furthermore, companies are starting to realise that ignoring supply chain-related animal welfare issues may create business and brand value risks [114,116], and this increases pressure on companies to include animal welfare as a focus of management. However, despite this pressure, the topic has remained a largely immature issue [117].

Until recently, investors regarded farm animal welfare as a niche ethical issue rather than a business issue and assumed that higher welfare farming inevitably results in higher financial costs for companies. Investors are an important influence on how companies manage the social and environmental impacts of their operations, and as such they can exert pressure on the behaviour of companies via their role as stakeholders or via the views they express when meeting with companies [118]. Some investor initiatives highlight the risks of intensive farming beyond animal welfare per se. These risks include threats to food safety, nutrition and public health (including antimicrobial resistance, the environment and labour rights, e.g., [119]). 

Tools such as the Business Benchmark on Farm Animal Welfare (BBFAW, an annual evaluation of the management of farm animal welfare in the world’s largest food companies) have helped investors to differentiate between those companies that manage farm animal welfare well and those that do not, as the BBFAW encourages improved reporting on farm animal welfare by companies and it provides a robust framework for assessing companies’ approaches to this issue [118]. There is evidence that companies respond to signals sent to them via responsible investors, as there has been a year on year increase in the overall BBFAW score for most of the businesses assessed, despite tightening of the Benchmark criteria and the increased emphasis on performance reporting and impact [11,120].

However, there is no room for complacency as about 47% of companies benchmarked in 2018 provided little or no information on their approach to farm animal welfare and this suggests that there is more to do, both in terms of encouraging improvements in policies, management systems and processes, and in ensuring that improvements are institutionalised and maintained over time [120].

To date, the BBFAW has not specifically assessed companies on policies requiring enrichment for pigs, but it does assess companies’ policies on routine mutilations. For pigs, these include tail docking, castration and teeth clipping. The proportion of companies that published policies in this area broadly increased over the benchmarked years (2012–2018). However, in 2018, only 24% of companies (with pigs in their supply) had made current (or future) public commitments specifically prohibiting the routine use of tail docking in pigs (data from [120] analysed). Furthermore, none of the companies accompanied commitments on tail docking with requirements for the provision of enrichment which is imperative if tail docking is not performed.

## 5. Conclusions

In the coming decades, the sustainability of pig production systems will not only rely on efficiency improvements at the herd level, but also on other factors: an increased use of alternative feed sources; reduced crude protein content in the rations; the proper use of pig manure as fertilizer through crop-livestock reconnection; the moderation of the human demand for pork; reducing the use of antibiotics [41,121]. We should add to that list the humane treatment of pigs and a focus on higher welfare production systems. This will involve extending the fundamental scientific basis for enrichment and building on what we already know and then keep channelling that knowledge to farms via advice and training.

With regards to regional progress, China is very much at the beginning of its pig welfare journey, against a background that the understanding of welfare in the general population is not particularly advanced. The USA has just passed the initial stages and is moving towards applying and generating animal welfare science and putting that into practice. The challenge for the US pig industry is to utilise the existing knowledge and extend it. The EU is well on its way on its welfare journey. However, the examples of issues on EU farms illustrate the challenge that the EC Pig Directive poses, especially with regards to the permanent provision of destructible materials. This requires continuous inspection and enforcement of the legislation. Table 3 summarises the current situation and likely future progress in the top pig-producing regions.

Designing effective enrichment is not an easy task, considering the range of aspects to consider in terms of design and presentation and the labour involved in implementing such a programme. Even when the guidance on effective enrichment has been followed (Section 2.1), the enrichment has to be assessed in situ to assess whether it is having the intended effect (e.g., by regular observations of the animals’ behaviour, such as aggression, displacements and vocalizations). This assessment should not only focus on short-term effects but monitor the long-term benefits of the enrichment strategy used, with a continuous cycle of evaluation and improvement. Producers may benefit from tailored advice that is specific to their situation [122] and the specific issues that they face on their farm and from the market they operate in. 

There is increasing pressure from the financial sector (and some of the published guidance includes the provision of enrichment for pigs [123] on food companies to include animal welfare as an area of focus within their business objectives, and this can lead to tangible change for animals on farms. Highlighting the economic benefits of effective enrichment and focusing on the returns of investment will help to break down barriers to further investment. 

The pig production community has had more than 50 years to implement and combine scientific and practical knowledge but is still a long way off reaching the ultimate destination of an enriched pig population.

## Figures and Tables

**Table 1:** The main characteristics of effective pig enrichment (from Van de Weerd [34]).

Main Characteristic	So That Pigs Can…	Provided in Such a Way That It…
Investigable	Explore the material with their nose (rooting) and mouth	Remains interesting to a pig (by providing sufficient quantities)
Manipulable	Change the material’s location, appearance and structure	Is accessible by suspending it at eye or floor level
Chewable (deformable, destructible)	Manipulate the material by biting and chewing	Is accessible for oral manipulation by all/most pigs in the pen
Edible (with an interesting texture, flavour or smell)	Ingest (eat) the material (that has some nutritional value) (Note: regular feed is not regarded as enrichment)	Is clean, safe and hygienic (minimising the risks of injury or contamination with chemicals or disease-causing agents)

**Table 2:** Sections of the EU Pig Directive that refer to enrichment materials [72].

Directive Section	Referring to:	Text
Article 3 (5.)	Sows and gilts	Member states shall ensure that, without prejudice to the requirements laid down in Annex I, sows and gilts have permanent access to manipulable material at least complying with the relevant requirements of that Annex.
Annex 1, Chapter 1 (4.)	All pigs	Notwithstanding Article 3 (5.), pigs must have permanent access to a sufficient quantity of material to enable proper investigation and manipulation activities, such as straw, hay, wood, sawdust, mushroom compost, peat or a mixture of such, which does not compromise the health of the animal.
Annex I, Chapter II: B. 3.	Sows and gilts	In the week before the expected farrowing time sows and gilts must be given suitable nesting material in sufficient quantity unless it is not technically feasible for the slurry system used in the establishment.
Annex I, Chapter II: C. 1.	Piglets	A part of the total floor, sufficient to allow the animals to rest together at the same time, must be solid or covered with a mat, or be littered with straw or any other suitable material.
Annex I, Chapter II: D. 3.	Weaners and rearing pigs	When signs of severe fighting appear, the causes shall be immediately investigated and appropriate measures taken, such as providing plentiful straw to the animals, if possible, or other materials for investigation. Animals at risk or particularly aggressive animals shall be kept separate from the group.

**Table 3:** Summary of the progress and likely future drivers for change in the journey towards implementing effective environmental enrichment for pigs in the World’s largest pig-producing regions (China, the European Union (EU) and the USA cover 77% of global production combined).

Region	Driver for Change			
	Regulation	Consumer/NGO Pressure	Guidelines/Assurance Schemes	Food Business/CSR Driven
USA	Some state restrictions on sow stalls and outright cruelty, no other federal or state legislation covering pig welfare during rearing. Unlikely to be a significant driver for change at the federal level but some progress may be made at the state level.	NGO pressure has focused on sow confinement with some progress and likely to extend to other pig welfare issues including enrichment. More information on consumer perceptions of unenriched environments and willingness to pay is needed.	Industry-led schemes exclude enrichment provision. A few third-party auditing and labelling schemes exist and include enrichment provision. Consumer demand for niche products is a relatively small but increasing market share.	Changing food business policy has been a significant driver for change in the case of sow stalls. In some cases, food business policy includes enrichment provision. Likely to be the biggest driver for change at scale in the future.
China	Legislation is minimal and mainly focuses on food safety rather than animal protection. Could be a driver for change in the future as the government approves of animal welfare activities.	Consumer awareness of animal welfare is low but increasing, particularly in large cities. NGOs are working on pig welfare and on increasing consumer awareness of pig welfare issues.	The CVMA ^a^ is developing non-binding animal welfare guidelines. ICCAW ^b^ pig welfare guidelines include enrichment. Third-party auditing and labelling schemes likely to appear in the future.	A few progressive businesses have their own pig welfare policies. Difficult to market products due to low consumer awareness. Likely to be a future driver with increasing awareness and interest in the BBFAW ^c^.
EU	Pigs are protected under a Directive that requires the provision of enrichment to all pigs. However, lack of adequate enrichment is a regular non-compliance to the Directive.	NGOs work both at the EU and country level to support the implementation of, or go beyond, minimum standards. European consumers appear more aware of farm animal welfare and the majority (82%) of those asked believed it should be better protected.	Private or voluntary initiatives promote pig welfare. These schemes increase compliance with legislation and drive welfare standards higher. Likely to increase in popularity and extend to a greater number of member states.	Many food businesses have progressive pig welfare policies to meet consumer expectations. Businesses, particularly supermarkets, can gain a competitive advantage. Financial institutions now also exerting pressure on food businesses to raise standards.

^a^ Chinese Veterinary Medical Association; ^b^ International Cooperation Committee on Animal Welfare; ^c^ Business Benchmark for Farm Animal Welfare.
